# Mental disorders, psychotropic drug dispensation and unfavourable sociodemographic factors in patients with myocardial infarction with and without obstructive coronary arteries^☆^

**DOI:** 10.1016/j.ijcrp.2026.200639

**Published:** 2026-04-17

**Authors:** Anna M. Nordenskjöld, Anders Wirén, Kerstin Welén Schef, Felicia Hakansson, Per Tornvall, Bertil Lindahl

**Affiliations:** a Department of Cardiology, Faculty of Medicine and Health, Örebro University, Örebro, Sweden; b Clinical Epidemiology and Biostatistics, Faculty of Medicine and Health, School of Medical Sciences, Örebro University, Örebro, Sweden; c Department of Clinical Sciences, Danderyd Hospital Division of Cardiology, Karolinska Institutet, Stockholm, Sweden; d Department of Clinical Science and Education, Karolinska Institutet, Södersjukhuset, Stockholm, Sweden; e Department of Medical Sciences and Uppsala Clinical Research Center, Uppsala University, Uppsala, Sweden

**Keywords:** Myocardial infarction, MINOCA, Mental disorders, Risk factors, Prognosis, Cohort study

## Abstract

**Background:**

Knowledge on the influence of mental disorders and unfavourable sociodemographic factors in patients experiencing myocardial infarction with non-obstructive coronary arteries (MINOCA) remains limited. We investigated the prevalence of mental disorders, psychotropic drug dispensation, and sociodemographic factors in patients with MINOCA and MI with coronary artery disease (MI-CAD) and assessed their association with prognosis.

**Methods and results:**

In this nationwide register-based cohort study of 8367 MINOCA and 109,059 MI-CAD patients, mental disorders (4.3% vs 2.6%, p < 0.001) and psychotropic drug dispensation (26.1% vs 17.4%, p < 0.001) were more frequent in MINOCA, particularly female patients. MINOCA patients were also more often divorced (21.1% vs 19.7%, p < 0.001), widowed (6.8% vs 4.9%, p < 0.001), or on sick leave (4.4% vs 3.2%, p < 0.001). Over a median 5.5-year follow-up, a major adverse cardiovascular event (MACE) occurred in 25.5% of MINOCA and 27.8% of MI-CAD patients (p < 0.001). Adjusted analyses showed that mental disorders and dispensed psychotropic drugs independently predicted MACE in both MINOCA (HR 1.27; 95% CI 1.15-1.40) and MI-CAD (HR 1.35; 95% CI 1.31-1.39).

**Conclusion:**

Mental disorders and psychotropic drug dispensation were more common in MINOCA than MI-CAD, particularly in female patients. Unfavourable sociodemographic factors were common in both groups, with a modest excess in MINOCA. The association between mental health variables and adverse outcomes in both conditions suggests shared mechanisms beyond traditional cardiovascular risk factors.

## Highlights

• Mental disorders and psychotropic drug dispensation are more prevalent during the six months prior MI in patients with MINOCA compared to MI-CAD.
• Unfavourable sociodemographic factors are more common in patients with MINOCA compared to MI-CAD at the time of MI.
• Mental disorders and psychotropic drug dispensation are independently associated with increased risk of major cardiovascular events during follow-up in both groups.
• Present and prior mental health conditions should be given greater consideration in the management of patients with myocardial infarction, particularly those with MINOCA, as they have a significant impact on prognosis.

## Graphical abstract

**Figure:**
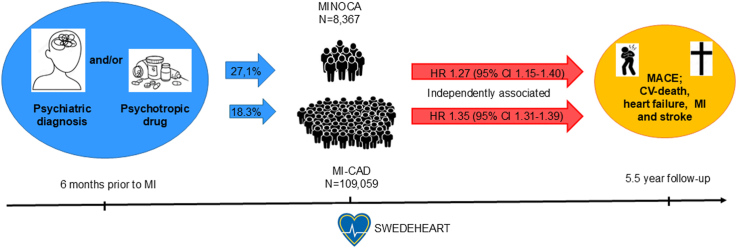

## 1 Introduction

The association of mental disorders such as mood disorders, psychosis, anxiety, and substance abuse with cardiovascular disease (CVD) is well-established [[1], [2], [3]]. The relationship is proposed to be bidirectional for at least some mental health disorders, as pre-existing depression increases the risk of incident CVD ^1,2,4-7^ and a history of CVD increases the risk of depression [1,2,4]. Mental disorders in patients with CVD are associated with an increased risk of CVD morbidity and mortality [1,2,5,6].

Psychosocial health factors including perceived stress, loss of control, loneliness, and critical life events are also associated with the development of CVD [1,2,7,8]. Psychosocial stress appears to be associated with a markedly increased risk of CVD like a diagnosis of serious mental disorder [6]. Work stress as well as unemployment and low socioeconomic status are also risk factors for CVD [1]. Sociodemographic factors such as marital status, educational level and occupational status may therefore represent valuable information for understanding a patient's risk profile.

Myocardial infarction with non-obstructive coronary arteries (MINOCA) constitutes approximately 6 -8% of all myocardial infarctions (MI) [9]. Patients with MINOCA are more likely to be younger, female, and with fewer comorbidities compared to patients with myocardial infarction due to coronary artery disease (MI-CAD) [10]. According to a large meta-analysis, the prognosis for patients with MINOCA is similar to that of MI-CAD patients with single/double-vessel coronary artery disease [11].

Studies with low numbers of participants have reported conflicting results, with higher [12], similar [[13], [14], [15], [16]] and lower [15] prevalence of mental disorders prior to, or at the time of, acute MI in MINOCA patients compared to MI-CAD patients.

As patients with MINOCA have fewer traditional CVD risk factors than those with MI-CAD but show similar unfavourable prognosis [2], one may hypothesize that non-traditional risk factors have a stronger impact in this group. The aim of the present study was to investigate the association of mental disorders, the dispensation of psychotropic drugs, and unfavourable sociodemographic factors with the development of acute MI and long-term prognosis in individuals with MINOCA and MI-CAD.

## 2 Methods

### 2.1 Study population

This nationwide descriptive cohort study was based on prospectively collected data from the Swedish Web-system for Enhancement and Development of Evidence-based care in Heart disease Evaluated According to Recommended Therapies (SWEDEHEART) registry [17].

**Fig. 1:**
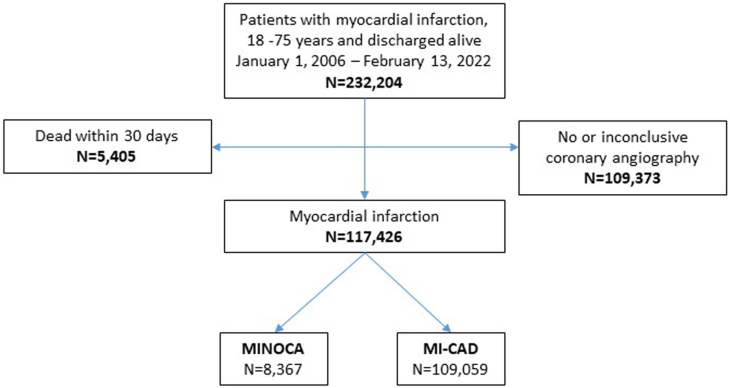
Flow chart of cohort selection.

All individuals admitted to a Swedish hospital due to acute MI and registered in the SWEDEHEART registry, from 1 January 2006 through 13 February 2022 were considered for inclusion. Exclusion criteria was age ≤18 years or ≥75 years at the time of MI, no in-hospital diagnostic coronary angiography, result of coronary angiography unknown, and death during hospitalization or within the 30 days following discharge. Coronary angiography during hospitalization revealing no stenosis or stenosis ≤50% of arterial diameter was identified as MINOCA. Patients who had previously undergone percutaneous coronary intervention or coronary artery bypass grafting were included in the MI-CAD group regardless of the findings of most recent coronary angiography. The study cohort consisted of 117,426 individuals of which 8367 were considered MINOCA and 109,059 MI-CAD (Fig. 1).

### 2.2 Follow-up and outcome

Follow-up started at discharge and continued until the occurrence of a major adverse cardiovascular event (MACE), death or the end of the study period, whichever occurred first. The primary outcome MACE was defined as a composite of CV-related death, re-hospitalization for MI, ischemic stroke, or heart failure. An additional secondary outcome was all-cause death.

Registration of an MI diagnosis within 30 days of discharge was not considered a new MI, based on ICD-10 coding criteria. The final day of follow-up was April 30, 2022.

### 2.3 Data sources

Data from the SWEDEHEART registry were merged with census data from Statistics Sweden and three mandatory Swedish population-based national registries: the Prescribed Drug Register, the Patient Register, and the Cause-of-death Register. The National Board of Health and Welfare warranted the compilation of data linked through the unique social security number of all residents of Sweden.

The SWEDEHEART registry provided patient medical history and in-hospital management.

Statistics Sweden provided sociodemographic data such as marital status, employment status, country of birth, and level of education.

The National Patient Register provided codes of diagnoses according to the International Classification of Diseases (ICD) for completed inpatient stays (nationwide since 1987) and specialised outpatient care (since 2001) as well as data of patients admitted to compulsory psychiatric care under the Compulsory Psychiatric Care Act (since 2010). Mental disorders registered 0 -6 months and 0 -12 months prior to MI were categorized as mental and behavioural disorders due to psychoactive substance use (ICD F10 -F19), schizophrenia, schizotypal and delusional disorders (ICD F20 -F29), affective disorders (ICD F31 -F33) and anxiety disorders (ICD F40 -F41).

The Swedish National Patient Register includes only diagnoses from inpatient and specialised outpatient care and not those from primary care, hence chiefly captures patients with complex illness.

The Swedish Prescribed Drug Register records complete data of prescribed drugs dispensed to individuals from all pharmacies in Sweden, regardless of whether the prescriber was from specialised or primary care and thus, reflects overall usage. Medications were defined according to the Anatomical Therapeutic Chemical (ATC) system. Five categories of psychotropic drugs in the six months prior to MI were recorded: antipsychotics (ATC code NO5A), anxiolytics (ATC code NO5B), hypnotics and sedatives (ATC code NO5C), antidepressants (ATC code NO6A), and psychostimulants (ATC code NO6B).

Data from the Cause of Death registry were used to investigate total mortality and CV-related mortality (ICD I00- I78).

According to Swedish law, no written informed consent is required for registration in the SWEDEHEART registry. Upon hospital admission, patients receive information about the registry, have the right to deny participation and may have their data erased upon request. The study was conducted according to the ethical guidelines of the 1975 Declaration of Helsinki and approved by the Regional Ethical Review Board (2012/60-31/2).

### 2.4 Statistical analysis

Descriptive statistics used counts and percentages for categorical variables and means with standard deviation (SD) and median with interquartile range (IQR) for continuous variables. To assess significance, Pearson's chi-squared test was used for categorical variables and Students t-test for continuous variables.

Cox regression analysis was used to investigate the prognostic value of traditional risk factors and selected non-traditional risk factors including mental health disorders, psychotropic drug dispensation, and negative sociodemographic factors by estimating the hazard ratio of MACE during follow-up. The traditional risk factors were selected from established cardiovascular risk factors ^10^. Smoking was used as a dichotomous variable; never versus current and previous.

Multivariable Cox regression was used to determine factors independently associated with MACE during follow-up. Analyses consisted of three models; one containing traditional CVD risk factors univariately associated with MACE, one with sociodemographic factors univariately associated with MACE and one that based on traditional CVD risk factors, sociodemographic factors and the presence of any mental disorder or psychotropic drug dispensation. Since it was missing in 20% and 18% of MINOCA and MI-CAD patients, respectively, the variable LDL-cholesterol was excluded from further analyses in the multivariable models despite its univariate association with MACE (Supplemental Table 1). The missing rate of the variable education level was 14% and 13% of MINOCA and MI-CAD, respectively. Given its potential informational value, and as data attrition was equivalent across both cohorts, with the missing information predominantly related to participants of immigrant origin, the variable was included in subsequent analyses.

As the gender ratio differs between the MINOCA and MI-CAD groups, outcome analyses were conducted separately for men and women. To examine the impact of year of diagnosis, outcomes before and after the start of 2017 (when a working group from the European Society of Cardiology proposed diagnostic criteria for MINOCA) were compared.

The significance level was set to a p-value of <0.05. IBM SPSS version 22.0 (IBM Corp., Armonk, NY) was used for statistical analyses.

## 3 Results

### 3.1 Baseline data

The study cohort consisted of 117,426 individuals of which 8367 had experienced MINOCA (7.1 %) and 109,059 MI-CAD (92.9 %). Compared to patients with MI-CAD, patients with MINOCA were younger, more often female, and had fewer traditional CVD risk factors including diabetes mellitus, smoking, hypercholesterolemia, and high body-mass-index (BMI). They were more likely to have been born in Sweden, had higher level of education, and were more frequently divorced or widowed and on sick leave or retired compared to MI-CAD patients (Table 1).

**Table 1:** Baseline characteristics.

Total, n	MINOCA	MI-CAD	p-value
	8367	109,059	
**Demographics**			
Age, y, median (IQR)	63.0 (55-70)	64.0 (57-70)	**<0.001**
Female, n (%)	5129 (61.3)	26,156 (24.0)	**<0.001**
Country of birth, n (%)			**<0.001**
Sweden	7019 (83.9)	88,304 (81.0)	
The rest of the Nordic countries	432 (5.2)	5600 (5.1)	
The rest of Europe	477 (5.7)	7596 (7.0)	
The rest of the world	439 (5.2)	7559 (6.9)	
Civil status, n (%)			**<0.001**
Married	4618 (55.4)	61,616 (56.7)	
Unmarried	1391 (16.7)	20,169 (18.6)	
Divorced	1755 (21.1)	21,436 (19.7)	
Widow/widower	568 (6.8)	5362 (4.9)	
Educational level, n (%)			**<0.001**
Elementary school	2202 (30.5)	32,147 (33.7)	
High school	3892 (53.9)	50,242 (52.6)	
University/Collage	1129 (15.6)	13,085 (12.0)	
Occupational status, n (%)			**<0.001**
Employed	3302 (42.3)	44,392 (43.6)	
Sick leave	342 (4.4)	3297 (3.2)	
Retired	3939 (50.4)	50,902 (50.0)	
Other	228 (2.9)	3208 (3.2)	
**Traditional risk factors**			
BMI kg/m^2^, median (IQR)	26.4 (23.6-29.8)	27.1 (24.7-30.1)	**<0.001**
Diabetes, n (%)	1006 (12.0)	20,765 (19.0)	**<0.001**
Chronic obstructive pulmonary disease, n (%)	721 (8.6)	4735 (4.3)	**<0.001**
Heart failure, n (%)	190 (2.3)	1883 (1.7)	**<0.001**
Hypertension, n (%)	3405 (40.7)	47,163 (43.3)	**<0.001**
Ischemic stroke, n (%)	309 (3.7)	4308 (4.0)	0.24
Smoking, n (%)			**<0.001**
Never	3569 (44.1)	37,319 (35.2)	
Previous	2779 (34.4)	35,613 (33.6)	
Present	1739 (21.5)	32,974 (31.1)	
Peripheral arterial disease, n (%)	120 (1.4)	2535 (2.3)	**<0.001**
**Laboratory findings**			
LDL-C mmol/L, mean (SD)	3.0 (1.0)	3.2 (1.1)	**<0.001**
Left ventricular ejection fraction, n (%)			**<0.001**
≥50%	5657 (79.6)	61,849 (64.7)	
40-49%	845 (11.9)	20,720 (21.7)	
30-39%	5.1 (6.0)	9843 (10.3)	
≤30%	172 (2.4)	3226 (3.4)	
**Medication, prior admission, n (%)**			
Aspirin	1135 (13.7)	16,942 (15.7)	**<0.001**
ACEI/ARB	2315 (27.9)	29,710 (27.6)	0.58
Beta blocker	1741 (21.0)	21,481 (20.0)	**0.027**
Statin	1425 (17.2)	20,370 (18.9)	**<0.001**
**Medication, at discharge, n (%)**			
Aspirin	7327 (87.6)	104,969 (96.3)	**<0.001**
ACEI/ARB	5223 (62.4)	89,000 (81.7)	**<0.001**
Beta blocker	6302 (75.3)	95,540 (87.7)	**<0.001**
Statin	7045 (84.2)	105,665 (97.0)	**<0.001**

ACEI, angiotensin converting enzyme inhibitor; ARB, angiotensin receptor blocker; BMI, body mass index; CABG. Coronary artery bypass grafting; LDL-C, low density lipoprotein cholesterol; MI, myocardial infarction; MINOCA, myocardial infarction with non-obstructive coronary arteries; MI-CAD, myocardial infarction and coronary artery disease; PCI, percutaneous coronary intervention.

### 3.2 Prevalence of psychiatric diagnoses and psychotropic drugs

Patients with MINOCA were more likely to have received a diagnosis of a mental or behavioural disorder resulting from psychoactive substance use, an affective disorder, or an anxiety disorder in the period six months prior to the index MI compared to those suffering MI-CAD (Table 2). The dispensing of antipsychotics, anxiolytics, antidepressants, hypnotics or sedatives was also higher in patients with MINOCA compared to MI-CAD. The prevalence of schizophrenia or a schizotypal or delusional disorder as well as dispensed prescription of a psychostimulants was similar in MINOCA and MI-CAD patients. A total of 2269 (27.1%) of MINOCA patients and 19,933 (18.3 %) had either received a diagnosis of a mental health disorder or had a psychotropic drug dispensed in the six months prior to the index MI (p < 0.001). In subgroup analysis stratified by sex, 1696 (33.1%) of female MINOCA patients and 7727 (29.5%) of female MI-CAD patients had either received a diagnosis of a mental health disorder or had a psychotropic drug dispensed in the six months prior to the index MI (p < 0.001). The corresponding proportion for men were 573 (17.7 %) in MINOCA and 12,206 (14.7%) in MI-CAD (p < 0.001). A total of 177 (2.1%) MINOCA patients and 1390 (1.3%) MI-CAD patients received a diagnosis of an affective disorder the year preceding MI (<0.001) (Supplemental Table 2).

**Table 2:** Mental disorders and dispensed psychotropic drugs six months prior of MI.

	MINOCA	MI-CAD	p-value
Mental disorders, n (%)			
Mental and behavioural disorders due to psychoactive substance use	178 (2.1)	1438 (1.3)	**<0.001**
Schizophrenia, schizotypal and delusional disorders	32 (0.4)	298 (0.3)	0.07
Affective disorders	124 (1.5)	968 (0.9)	**<0.001**
Anxiety	91 (1.1)	561 (0.5)	**<0.001**
Any psychiatric diagnosis	358 (4.3)	2869 (2.6)	**<0.001**
**Psychotropic drugs, n (%)**			
Antipsychotics (NO5A)	150 (1.8)	1475 (1.4)	**<0.001**
Anxiolytics (NO5B)	764 (9.1)	5664 (5.2)	**<0.001**
Hypnotics and sedatives (NO5C)	1173 (14.0)	9992 (9.2)	**<0.001**
Antidepressants (NO6A)	1124 (13.4)	9877 (9.1)	**<0.001**
Psychostimulants (NO6B)	32 (0.4)	304 (0.3)	0.087
Any psychotropic drug	2184 (26.1)	18,990 (17.4)	**<0.001**
**Any mental disorder or drug, n (%)**	2269 (27.1)	19,933 (18.3)	**<0.001**

MINOCA, myocardial infarction with non-obstructive coronary arteries; MI-CAD, myocardial infarction and coronary artery disease.

### 3.3 The association with traditional CVD risk factors, psychological factors, and prognosis

During a median follow-up time of 5.5 years, 32,430 patients experienced a MACE, 2131 (25.5%) in the MINOCA group and 30,299 (27.8%) in the MI-CAD group (p = 0.001). A total of 20,145 patients died (all-cause death): 1466 (17.5%) MINOCA patients and 18,679 (17.1%) MI-CAD patients (p = 0.357). 7196 deaths were CV-related: 408 (4.9%) MINOCA and 6788 (6.2%) MI-CAD patients (p < 0.001). The primary cause of death was related to a psychiatric diagnosis in 43 patients (ICD F00-F99), all MI-CAD patients (p = 0.69). No patient died due to intentional self-harm (ICD X60-X84).

The traditional CVD risk factors age, diabetes mellitus, sex, hypertension, LDL-cholesterol level, and smoking were associated with MACE in both MINOCA and MI-CAD patients, whereas BMI were associated with MACE in MI-CAD patients alone. Civil status, educational level, occupation status, the presence of a mental disorder, and a psychotropic drug were associated with MACE in both MINOCA and MI-CAD patients. Country of birth was associated with MACE in MI-CAD patients alone (Supplemental Table 3).

In the multivariable analysis, a recent mental disorder or a recently dispensed psychotropic drug, age, diabetes, sex, hypertension, smoking, civil status, education level, and occupation status were each independently associated with MACE in both MINOCA and MI-CAD patients (Table 3). Additionally, in patients with MI-CAD the country of birth was also independently associated with MACE (Table 3).

**Table 3:** Risk factors independently associated with MACE. The analyses were made in three steps; the first model contained traditional risk factors, the second model contained sociodemographic factors, and the third model contained both traditional risk factors, sociodemographic factors and the presence of any mental disorder or psychotropic drug.

MINOCA	HR	95 % CI	p-value	HR	95 % CI	p-value	HR	95 % CI	p-value
Traditional risk factors									
Age	1.05	1.05-1.06	**<0.001**				1.04	1.03-1.04	**<0.001**
Diabetes	1.58	1.40-1.78	**<0.001**				1.52	1.35-1.71	**<0.001**
Sex^1^	0.81	0.74-0.88	**<0.001**				0.73	0.67-0.81	**<0.001**
Hypertension	1.15	1.05-1.26	**<0.001**				1.14	1.04-1.25	**0.005**
Smoking^2^	1.38	1.26-1.51	**<0.001**				1.30	1.19-1.42	**<0.001**
**Sociodemographics**									
Civil status^3^				1.30	1.19-1.43	**<0.001**	1.25	1.13-1.37	**<0.001**
Educational level^4^				0.78	0.71–0.85	**<0.001**	0.85	0.78-0.94	**<0.001**
Occupational status^5^				2.27	2.05-2.51	**<0.001**	1.52	1.34-1.72	**<0.001**
**Any disorder or drug**							1.27	1.15-1.40	**<0.001**
**MI-CAD**	**HR**	**95 % CI**	**p-value**	**HR**	**95 % CI**	**p-value**	**HR**	**95 % CI**	**p-value**
**Traditional risk factors**									
Age	1.04	1.04-1.04	**<0.001**				1.03	1.02-1.03	**<0.001**
BMI	1.00	1.00-1.01	**<0.001**				1.00	1.00-1.01	**<0.001**
Diabetes	1.77	1.72–1.82	**<0.001**				1.71	1.66-1.76	**<0.001**
Sex^1^	1.05	1.02-1.08	**<0.001**				0.96	0.93-0.98	**0.002**
Hypertension	1.27	1.24–1.30	**<0.001**				1.25	1.22-1.28	**<0.001**
Smoking^2^	1.28	1.24-1.31	**<0.001**				1.22	1.18-1.25	**<0.001**
**Sociodemographics**									
Country of birth^6^				1.06	1.03-1.09	**<0.001**	1.08	1.05-1.12	**<0.001**
Civil status^3^				1.24	1.21-1.27	**<0.001**	1.16	1.13-1.19	**<0.001**
Educational level^4^				0.84	0.82-0.86	**<0.001**	0.88	0.86-0.91	**<0.001**
Occupational status^5^				1.87	1.83-1.92	**<0.001**	1.35	1.31-1.40	**<0.001**
**Any disorder or drug**							1.35	1.31-1.39	**<0.001**

1. Men vs women. 2. Never vs previous/current smoker. 3. Sweden vs other countries. 4. Married/single vs divorced/widowed. 5. Elementary school vs higher education. 6. Employed vs sick leave/retired/other. MINOCA, myocardial infarction with non-obstructive coronary arteries; MI-CAD, myocardial infarction and coronary artery disease; HR, hazard ratio; CI, confidence interval.

In sex-specific subgroup analyses, a recent mental disorder or a recently dispensed psychotropic drug remained independently associated with MACE in both men and women, irrespective of MINOCA or MI-CAD status (Supplemental Table 4).

In analyses stratified by year of diagnosis (before vs. after 2017), the prevalence of a recent mental disorder or a recently dispensed psychotropic drug was higher among patients diagnosed after 2017 in both groups (before 2017: MI-CAD 17.0%, MINOCA 26.1%; after 2017: MI-CAD 18.8%, MINOCA 27.6%; p < 0.001 for both). Moreover, these factors remained independently associated with MACE across both time periods (Supplemental Table 5).

With respect to all-cause death, univariate analyses were made of the exposures 1) any mental disorder or 2) any psychotropic drug, and 3) the combined variable any mental disorder or psychotropic drug. In MINOCA patients, the respective associations with all-cause death were HR 2.36 (95% CI 1.94 -2.87), HR 1.73 (95% CI 1.55 -1.92), HR 1.75 (1.58 -1.95) and in MI-CAD patients HR 2.01 (95% CI 1.87 -2.16), HR 1.84 (95% CI 1.78 -1.90), HR 1.86 (1.80 -1.92).

## 4 Discussion

This nationwide observational registry-based cohort study is the first to our knowledge to demonstrate that individuals experiencing MINOCA show significantly higher prevalence of mental disorders, have more psychotropic drugs dispensed and have more unfavourable sociodemographic factors than patients with MI-CAD. We were also able to demonstrate an association between these factors and adverse outcomes in both MINOCA and MI-CAD. The majority of MINOCA patients were female, whereas a male predominance was noted among MI-CAD patients. Other differences in sociodemographic factors were modest in magnitude yet reached statistical significance owing to the large patient cohorts. In subgroup analyses stratified by sex, the prevalence of diagnosed mental health disorders or dispensed psychotropic medications in the six months preceding the index MI was higher in women than in men; however, the difference between MINOCA and MI-CAD persisted.

Mental and behavioural disorders due to psychoactive substance use, affective disorders and anxiety disorders were more prevalent in MINOCA patients compared to MI-CAD, although numbers were low in both groups. The psychiatric diagnoses in the present study were derived from the Swedish National Patient Register, and included only diagnoses from inpatient care and specialised outpatient care and did not include diagnoses from primary care. The relatively low reports of, for example depression was therefore expected, as the registry mainly captures patients with complex illness requiring treatment in-hospital or at specialised outpatient clinics. Nevertheless, the prevalence of mental disorders with serious symptoms were higher in patients with MINOCA than in patients with MI-CAD both six and 12 months prior to MI. The prevalence of depression diagnosed in-hospital or in specialised outpatient care in the 12 months preceding MI was 1.3 % in the present study, which can be compared to the pooled annual incidence of major depressive disorder reported to be approximately 3.0% both globally and in Scandinavia [[18], [19]]. As the aim of the study was to investigate the association of current mental disorders with the development of acute MI, we opted to use only data from the six months prior to MI.

In Sweden, most individuals with mental disorders and mental health conditions are diagnosed and treated within primary care. We therefore used the dispensing of medications used in mental disorders and in related mental health conditions, to estimate the overall prevalence of psychiatric morbidity. The dispensing of antidepressants, hypnotics and sedatives, and anxiolytics was high and significantly more common among MINOCA patients than in MI-CAD patients. This finding agrees with a smaller Swedish study in which the prevalence of mental disorders including depression, anxiety disorder, sleep disorder, bipolar disorder, anorexia, social phobia, attention deficit disorder, and chronic fatigue syndrome extracted from medical records was significantly higher in MINOCA patients (20%) than in matched MI-CAD patients (11%) and matched controls without CVD (3%) [20]. In the same study, 59% of the MINOCA patients reported physical and/or mental stress in the seven days prior to admission compared to 13% of the MI-CAD patients [21]. A small Spanish study reported that 28% of MINOCA patients compared to 13% of MI-CAD patients had experienced previous mental disorder, and 71% of MINOCA compared to 32% of MI-CAD acknowledged emotional stress [22].

A recent mental disorder diagnosed in-hospital or in specialised outpatient care was associated with both all-cause and CV-related death and MACE in both MINOCA and MI-CAD patients which agrees with previous studies demonstrating increased mortality post-MI in patients with severe mental illness (e.g. bipolar disorder or schizophrenia) and depression [5,6]. The association of a mental disorders in MINOCA patients with poor prognosis is supported by a small Chinese study in which MINOCA patients, initially diagnosed with depression according to the 17-item Hamilton depression score during hospitalization exhibited a significantly increased risk of a new CV-event and of all-cause mortality [23].

There are no previous reports of relatively poor prognosis in patients with MINOCA receiving therapy with antipsychotics, anxiolytics, antidepressants, hypnotics, or sedatives. However, our results with respect to anxiety disorders inferred from the use of anxiolytics are in accordance with the outcome in a small Chinese study, which found anxiety diagnosed in the seven days preceding MINOCA to be significantly associated with increased risk of MACE and all-cause mortality during follow-up [24].

In the present study, the prevalence of mental disorders and psychotropic drugs, within six months of MI, was higher among MINOCA patients than among MI-CAD patients and was associated with adverse prognosis in both. The difference also remained in subgroup analyses stratified by sex. Unfavourable sociodemographic factors were marginally, but statistically significant, more commonly found in MINOCA compared to MI-CAD and also associated with adverse prognosis. This finding indicating a common underlying mechanism beyond the traditional CVD risk factors agrees with reports of associations with both mental disorders [[1], [2], [3], [4], [5], [6], [25], [26], [27]] and negative psychosocial health factors [1,2,7,8]. Exposure to acute or chronic stress may result in altered neurochemical function, such as disruptions in the synthesis or activity of serotonin and dopamine, which, in turn, may influence mood and the risk of CVD ^28^. Chronic dysregulation of autonomic functions, such as imbalance between the sympatric and parasympathetic systems, is proposed to be an important mechanism linking depression to CVD risk and adverse cardiovascular events [28]. Additionally, chronic inflammation, endothelial dysfunction and increased platelet activation and thrombosis are other mechanisms that occurs in both depression and CVD and may contribute significantly to the association between the conditions [29]. Furthermore, shared genetic factors influencing both major depressive disorders and CVD have shown to be associated with traditional CVD risk factors such as diabetes mellitus and smoking, as well as with loneliness [30].

A possible explanation for the association between psychiatric comorbidity and poor prognosis in the present study may be insufficient adherence to secondary preventive pharmacological therapy and lifestyle modifications recommended in guidelines, as demonstrated in previous studies [[31], [32], [33]].

### 4.1 Strengths and limitations

The major strengths of this nationwide registry-based study lie in the large study cohort and the unbiased selection. Virtually all patients in Sweden with acute MI who underwent coronary angiography during hospitalization over the course of 15 years are included, increasing the reliability and generalizability of the study results. The use of a registry reduces potential selection bias associated with studies of patients at selected hospitals or enrolled in health care insurance systems. The SWEDEHEART registry and the mandatory national registries have high coverage and data validity [17,34].

The registry-based analysis also has several limitations: (1) the analysis relied on ICD-codes, and the possibility of coding errors cannot be ruled out. (2) As diagnostic criteria for MINOCA were not proposed until 2017, it is difficult to estimate how many patients currently meeting criteria for MINOCA were diagnosed with a non-MI related condition. However, patients diagnosed after 2017 had a similar or higher prevalence of psychiatric comorbidity and dispensed psychotropic medications than patients diagnosed before 2017, and the association with MACE remained. (3) Cardiac magnetic resonance imaging was seldom used in patients with suspected MINOCA during the study period, and it is therefore likely that some of the patients labelled as MI in this study had an undiagnosed myocarditis or takotsubo syndrome. (4) The number of patients in the study population with type 2 MI are unknown. The ICD-code for type 2 MI (I21.A1) was not introduced until October 2018 and was therefore not useful in this retrospective study. There are multiple possible causes for the oxygen demand/supply discrepancy in the myocardial cells resulting in type 2 MI (e.g. anaemia, tachy- and bradyarrhythmia, hypertension, hypotension, hypoxia, coronary spasm). Information sufficient to allow us to adjust for all these factors in a systematic way was not available in the registries. A previous diagnosis of heart failure or COPD or a record of atrial fibrillation at admission is not more strongly associated with type 2 MI than with type 1 MI. We therefore opted to describe the cohorts in more detail (Table 1) instead of excluding patients.

The diagnoses of mental disorders in the present study were derived from the Swedish National Patient Register, and thus included only diagnoses from inpatient care and specialised outpatient care. The true numbers of patients with less complex mental health disorders are therefore unknown. Although data of dispensed antipsychotics, anxiolytics, antidepressants, psychostimulants, hypnotics, and sedatives from the Swedish Prescribed Drug registry are helpful to estimate the prevalence of less complex psychiatric comorbidity, the register does not contain information of the underlying diagnoses motivating the prescribing of the drugs. Furthermore, it is unknown how many patients who picked up the prescribed medication from the pharmacy never actually took it. It is also unknown how many patients experienced mental health issues but remained undiagnosed or were treated with non-pharmacological psychological interventions, such as cognitive behavioural therapy.

### 4.2 Conclusion

The prevalence of mental disorders and dispensed psychotropic drugs was more common in patients with MINOCA than in patients with MI-CAD, particularly female patients. Unfavourable sociodemographic factors were frequently observed in both groups, with a slight predominance among patients with MINOCA. The association between these factors in both MINOCA and MI-CAD may indicate a common underlying mechanism beyond traditional CVD risk factors.

## CRediT authorship contribution statement

**Anna M. Nordenskjöld:** Writing – original draft, Visualization, Supervision, Resources, Project administration, Methodology, Investigation, Funding acquisition, Formal analysis, Conceptualization. **Anders Wirén:** Writing – review & editing, Methodology, Formal analysis. **Kerstin Welén Schef:** Writing – review & editing, Methodology. **Felicia Hakansson:** Writing – review & editing, Methodology. **Per Tornvall:** Writing – review & editing, Methodology. **Bertil Lindahl:** Writing – review & editing, Methodology.

## Declaration of competing interest

None.
