# Supplementary material for: Mental disorders, psychotropic drug dispensation and unfavourable sociodemographic factors in patients with myocardial infarction with and without obstructive coronary arteries
Source: Int J Cardiol Cardiovasc Risk Prev. 2026 Apr 17;29:200639. doi: 10.1016/j.ijcrp.2026.200639 (PMC13123369; doi:10.1016/j.ijcrp.2026.200639)
Supplement: Multimedia component 1 [file mmc1.docx]

**Supplemental table 1. Missing variables.**

|  | **MINOCA** | **MI-CAD** |
| --- | --- | --- |
| Total, n | 8,367 | 109,059 |
| **Demographics** |  |  |
| Age | 0 | 0 |
| Gender | 0 | 0 |
| Country of birth | 0 | 0 |
| Civil status | 35 | 476 |
| Educational level | 1,144 | 13,585 |
| Occupational status | 556 | 7,260 |
| **Traditional risk factors** |  |  |
| BMI | 806 | 9,391 |
| Diabetes | 0 | 0 |
| Chronic obstructive pulmonary disease | 0 | 0 |
| Heart failure | 0 | 0 |
| Hypertension | 525 | 35 |
| Ischemic stroke | 0 | 0 |
| Smoking | 280 | 3,153 |
| Peripherial arterial disease | 0 | 0 |
| Previous PCI or CABG | 0 | 0 |
| **Laboratory findings** |  |  |
| LDL-C | 1,699 | 19,267 |
| Left ventricular ejection fraction | 1,264 | 13,421 |
| **Medication, prior admission** |  |  |
| Aspirin | 59 | 1,237 |
| ACEI/ ARB | 65 | 1,411 |
| Beta blocker | 69 | 1,517 |
| Statin | 58 | 1,251 |
| **Medication, at discharge** |  |  |
| Aspirin | 4 | 87 |
| ACEI/ARB | 0 | 70 |
| Beta blocker | 2 | 84 |
| Statin | 1 | 99 |

ACEI, angiotensin converting enzyme inhibitor; ARB, angiotensin receptor blocker; BMI, body mass index; CABG. Coronary artery bypass grafting; LDL-C, low density lipoprotein cholesterol; MI, myocardial infarction; MINOCA, myocardial infarction with non-obstructive coronary arteries; MI-CAD, myocardial infarction and coronary artery disease; PCI, percutaneous coronary intervention.
