# Supplementary material for: Mental disorders, psychotropic drug dispensation and unfavourable sociodemographic factors in patients with myocardial infarction with and without obstructive coronary arteries
Source: Int J Cardiol Cardiovasc Risk Prev. 2026 Apr 17;29:200639. doi: 10.1016/j.ijcrp.2026.200639 (PMC13123369; doi:10.1016/j.ijcrp.2026.200639)
Supplement: Multimedia component 2 [file mmc2.docx]

**Supplemental Table 2.** Mental disorders 12 months prior MI.

| **Number (%)** | **MINOCA** | **MI-CAD** | **p-value** |
| --- | --- | --- | --- |
| Mental and behavioural disorders due to psychoactive substance use | 225 (2.7) | 1921 (1.8) | **<0.001** |
| Schizophrenia, schizotypal and delusional disorders | 41 (0.5) | 410 (0.4) | 0.07 |
| Affective disorders | 177 (2.1) | 1390 (1.3) | **<0.001** |
| Anxiety | 142 (1.7) | 829 (0.8) | **<0.001** |
| Any psychiatric diagnosis | 470 (5.6) | 3892 (3.6) | **<0.001** |

MINOCA, myocardial infarction with non-obstructive coronary arteries; MI-CAD, myocardial infarction and coronary artery disease.
