# Supplementary material for: Mental disorders, psychotropic drug dispensation and unfavourable sociodemographic factors in patients with myocardial infarction with and without obstructive coronary arteries
Source: Int J Cardiol Cardiovasc Risk Prev. 2026 Apr 17;29:200639. doi: 10.1016/j.ijcrp.2026.200639 (PMC13123369; doi:10.1016/j.ijcrp.2026.200639)
Supplement: Multimedia component 3 [file mmc3.docx]

**Supplemental table 3.**  Traditional and untraditional risk factors univariate associated with MACE in patients with MINOCA and MI-CAD presented with hazard ratios (HRs) and 95% confidence intervals (CIs)

|  |  | **MINOCA HR** | **95 % CI** | **p-value** | **MI-CAD HR** | **95 % CI** | **p-value** |
| --- | --- | --- | --- | --- | --- | --- | --- |
| **Traditional risk factors** |  |  |  |  |  |  |  |
| Age |  | 1.05 | 1.05-1.06 | **<0.001** | 1.04 | 1.04-1.04 | **<0.001** |
| BMI |  | 1.00 | 1.00-1.01 | 0.446 | 1.01 | 1.01-1.01 | **<0.001** |
| Diabetes |  | 1.88 | 1.68-2.10 | **<0.001** | 1.97 | 1.92-2.02 | **<0.001** |
| Sex^1^ |  | 0.92 | 0.84-1.00 | **0.045** | 1.14 | 1.11-1.17 | **<0.001** |
| Hypertension |  | 1.44 | 1.32-1.57 | **<0.001** | 1.56 | 1.52-1.60 | **<0.001** |
| LDL-cholesterol |  | 0.83 | 0.79-0.87 | **<0.001** | 0.79 | 0.78-0.80 | **<0.001** |
| Smoking^2^ |  | 1.36 | 1.24-1.48 | **<0.001** | 1.22 | 1.19-1.25 | **<0.001** |
| **Sociodemographic factors** |  |  |  |  |  |  |  |
| Country of birth^3^ |  | 0.99 | 0.88-1.12 | 0.914 | 1.07 | 1.04-1.10 | **<0.001** |
| Civil status^4^ |  | 1.44 | 1.32-1.58 | **<0.001** | 1.31 | 1.27-1.34 | **<0.001** |
| Educational level^5^ |  | 0.69 | 0.63-0.75 | **<0.001** | 0.76 | 0.75-0.78 | **<0.001** |
| Occupation status^6^ |  | 2.42 | 2.19-2.67 | **<0.001** | 1.94 | 1.90-1.99 | **<0.001** |
| Any mental disorder |  | 1.56 | 1.29-1.89 | **<0.001** | 1.65 | 1.55-1.78 | **<0.001** |
| Any psychotropic drug | | 1.40 | 1.28-1.53 | **<0.001** | 1.52 | 1.48-1.56 | **<0.001** |
| Any disorder or drug |  | 1.41 | 1.29-1.55 | **<0.001** | 1.54 | 1.50-1.58 | **<0.001** |

1. Men vs women. 2. Never vs previous/current smoker. 3. Sweden vs other countries. 4. Married/single vs divorced/widowed. 5. Elementary school vs higher education. 6. Employed vs sick leave/retired/other. BMI, body mass index; LDL, low density lipoprotein; MINOCA, myocardial infarction with non-obstructive coronary arteries; MI-CAD, myocardial infarction and coronary artery disease.
