# Supplementary material for: Mental disorders, psychotropic drug dispensation and unfavourable sociodemographic factors in patients with myocardial infarction with and without obstructive coronary arteries
Source: Int J Cardiol Cardiovasc Risk Prev. 2026 Apr 17;29:200639. doi: 10.1016/j.ijcrp.2026.200639 (PMC13123369; doi:10.1016/j.ijcrp.2026.200639)
Supplement: Multimedia component 4 [file mmc4.docx]

**Supplemental table 4.** Risk factors independently associated with MACE in multivariable analysis stratified by sex.

|  |  | **MINOCA women** |  |  | **MI-CAD women** |  |
| --- | --- | --- | --- | --- | --- | --- |
| **Traditional risk factors** | **HR** | **95% CI** | **p-value** | **HR** | **95% CI** | **p-value** |
| Age | 1.04 | 1.03-1.05 | **<0.001** | 1.03 | 1.03-1.03 | **<0.001** |
| Diabetes | 1.50 | 1.28-1.76 | **<0.001** | 1.87 | 1.78-1.97 | **<0.001** |
| Hypertension | 1.08 | 0.96-1.22 | 0.185 | 1.23 | 1.17-1.29 | **<0.001** |
| Smoking^1^ | 1.32 | 1.18-1.48 | **<0.001** | 1.20 | 1.14-1.26 | **<0.001** |
| **Sociodemographic factors** |  |  |  |  |  |  |
| Civil status^2^ | 1.27 | 1.13-1.42 | **<0.001** | 1.12 | 1.06-1.17 | **<0.001** |
| Educational level^3^ | 0.81 | 0.72-0.92 | **<0.001** | 0.88 | 0.84-0.92 | **<0.001** |
| Occupational status^4^ | 1.28 | 1.08-1.53 | **0.005** | 1.44 | 1.34-1.54 | **<0.001** |
| **Any disorder or drug** | 1.22 | 1.09-1.38 | **<0.001** | 1.29 | 1.23-1.36 | **<0.001** |
|  |  | **MINOCA men** |  |  | **MI-CAD men** |  |
| **Traditional risk factors** | **HR** | **95% CI** | **p-value** | **HR** | **95% CI** | **p-value** |
| Age | 1.03 | 1.02-1.04 | **<0.001** | 1.02 | 1.02-1.03 | **<0.001** |
| Diabetes | 1.56 | 1.30-1.86 | **<0.001** | 1.66 | 1.61-1.72 | **<0.001** |
| Hypertension | 1.23 | 1.06-1.43 | **0.005** | 1.25 | 1.22-1.29 | **<0.001** |
| Smoking^1^ | 1.28 | 1.11-1.48 | **0.001** | 1.23 | 1.19-1.27 | **<0.001** |
| **Sociodemographic factors** |  |  |  |  |  |  |
| Civil status^2^ | 1.19 | 1.01-1.40 | **0.038** | 1.19 | 1.16-1.23 | **<0.001** |
| Educational level^3^ | 0.91 | 0.78-1.05 | 0.193 | 0.88 | 0.86-0.91 | **<0.001** |
| Occupational status^4^ | 1.81 | 1.51-2.16 | **<0.001** | 1.35 | 1.30-1.39 | **<0.001** |
| **Any disorder or drug** | 1.03 | 1.02-1.04 | **<0.001** | 1.39 | 1.34-1.44 | **<0.001** |

1. Never vs previous/current smoker. 2. Married/single vs divorced/widowed. 3. Elementary school vs higher education. 4. Employed vs sick leave/retired/other. MINOCA, myocardial infarction with non-obstructive coronary arteries; MI-CAD, myocardial infarction and coronary artery disease; HR, hazard ratio; CI, confidence interval.
