# Supplementary material for: Mental disorders, psychotropic drug dispensation and unfavourable sociodemographic factors in patients with myocardial infarction with and without obstructive coronary arteries
Source: Int J Cardiol Cardiovasc Risk Prev. 2026 Apr 17;29:200639. doi: 10.1016/j.ijcrp.2026.200639 (PMC13123369; doi:10.1016/j.ijcrp.2026.200639)
Supplement: Multimedia component 5 [file mmc5.docx]

**Supplemental table 5.** Risk factors independently associated with MACE in multivariable analysis stratified by year of diagnosis (before/after 2017).

|  |  | **MI-CAD before 2017, n=74.423** | | | | |  |  | **MI-CAD after 2017, n=34.636** | | |  | |
| --- | --- | --- | --- | --- | --- | --- | --- | --- | --- | --- | --- | --- | --- |
|  | **HR** | | | **95 % CI** | **p-value** | | | **HR** | | **95 % CI** | **p-value** | | |
| Age | 1.03 | | | 1.02-1.03 | **<0.001** | | | 1.02 | | 1.01-1.02 | **<0.001** | | |
| Diabetes | 1.71 | | | 1.66-1.77 | **<0.001** | | | 1.77 | | 1.65-1.89 | **<0.001** | | |
| Gender | 0.93 | | | 0.90-0.95 | **<0.001** | | | 1.02 | | 0.95-1.09 | 0.571 | | |
| Hypertension | 1.25 | | | 1.21-1.28 | **<0.001** | | | 1.33 | | 1.24-1.42 | **<0.001** | | |
| Smoking^1^ | 1.22 | | | 1.19-1.26 | **<0.001** | | | 1.19 | | 1.11-1.27 | **<0.001** | | |
| Civil status^2^ | 1.17 | | | 1.14-1.21 | **<0.001** | | | 1.15 | | 1.08-1.23 | **<0.001** | | |
| Educational level^3^ | 0.89 | | | 0.87-0.92 | **<0.001** | | | 0.89 | | 0.83-0.95 | **<0.001** | | |
| Occupational status^4^ | 1.37 | | | 1.32-1.41 | **<0.001** | | | 1.29 | | 1.19-1.41 | **<0.001** | | |
| **Any disorder or drug** | 1.34 | | | 1.30-1.39 | **<0.001** | | | 1.44 | | 1.34-1.54 | **<0.001** | | |
|  |  | | **MINOCA before 2017, n=6.032** | | |  | |  | **MINOCA after 2017, n= 2.335** | | | |  |
| Age | 1.04 | | | 1.03-1.04 | **<0.001** | | | 1.03 | | 1.01-1.05 | **0.002** | | |
| Diabetes | 1.52 | | | 1.34-1.73 | **<0.001** | | | 1.43 | | 1.00-2.05 | 0.051 | | |
| Gender | 0.73 | | | 0.67-0.81 | **<0.001** | | | 0.71 | | 0.54-0.94 | **0.016** | | |
| Hypertension | 1.14 | | | 1.04-1.26 | **0.007** | | | 1.23 | | 0.94-1.63 | 0.137 | | |
| Smoking^1^ | 1.28 | | | 1.16-1.41 | **<0.001** | | | 1.47 | | 1.11-1.94 | **0.008** | | |
| Civil status^2^ | 1.25 | | | 1.13-1.38 | **<0.001** | | | 1.19 | | 0.90-1.59 | 0.226 | | |
| Educational level^3^ | 0.88 | | | 0.80-0.97 | **0.011** | | | 0.74 | | 0.55-1.01 | 0.055 | | |
| Occupational status^4^ | 1.55 | | | 1.36-1.77 | **<0.001** | | | 1.24 | | 0.85-1.81 | 0.260 | | |
| **Any disorder or drug** | 1.24 | | | 1.11-1.37 | **<0.001** | | | 1.54 | | 1.16-2.04 | **0.002** | | |

1. Never vs previous/current smoker. 2. Married/single vs divorced/widowed. 3. Elementary school vs higher education. 4. Employed vs sick leave/retired/other. MINOCA, myocardial infarction with non-obstructive coronary arteries; MI-CAD, myocardial infarction and coronary artery disease; HR, hazard ratio; CI, confidence interval.
